# Sec translocon has an insertase-like function in addition to polypeptide conduction through the channel

**DOI:** 10.12688/f1000research.21065.1

**Published:** 2019-12-20

**Authors:** Koreaki Ito, Naomi Shimokawa-Chiba, Shinobu Chiba

**Affiliations:** 1 Faculty of Life Sciences and Institute for Protein Dynamics, Kyoto Sangyo University, Kyoto, Japan

**Keywords:** sec translocon, insertase, SecY, Sec61, membrane protein, YidC

## Abstract

The Sec translocon provides a polypeptide-conducting channel, which is insulated from the hydrophobic lipidic environment of the membrane, for translocation of hydrophilic passenger polypeptides. Its lateral gate allows a downstream hydrophobic segment (stop-transfer sequence) to exit the channel laterally for integration into the lipid phase. We note that this channel model only partly accounts for the translocon function. The other essential role of translocon is to facilitate *de novo* insertion of the N-terminal topogenic segment of a substrate polypeptide into the membrane. Recent structural studies suggest that *de novo* insertion does not use the polypeptide-conducting channel; instead, it takes place directly at the lateral gate, which is prone to opening. We propose that the *de novo* insertion process, in concept, is similar to that of insertases (such as YidC in bacteria and EMC3 in eukaryotes), in which an intramembrane surface of the machinery provides the halfway point of insertion.

## Introduction

In protein localization, hydrophobic segments of polypeptides play a central role by their ability to partition into the hydrophobic core of the membrane ^1– 3^. At the same time, proteins that are integrated into the membrane or localized in extra-cytosolic locations require facilitation by specific cellular mechanisms in reaching their destinations and achieving their topographical relationships to the membrane. The difficulties arise because the cytosol, where the translation of genetic messages takes place, is compartmentalized by membranes to make it discrete from other cellular compartments and the surroundings. Consequently, newly synthesized polypeptides must follow a proper pathway typically involving bio-machinery to integrate into or cross the membrane. Such pathways alleviate the difficulty of overcoming specific energy barriers that the moving polypeptide encounters. Hydrophilic parts of polypeptides must overcome the hydrophobic barrier of the lipid hydrocarbon to cross the membrane, whereas hydrophobic polypeptide segments should overcome the hydrophilic barrier of the phospholipid head groups to partition into the lipidic membrane interior.

In this review, we attempt to clarify the role of the Sec translocon, a principal and conserved cellular machine, in assisting protein translocation across and insertion into the membrane, corresponding to the cytoplasmic membrane in the case of bacteria and the endoplasmic reticulum (ER) membrane in the case of eukaryotic cells ^4^. Specifically, we illuminate the importance of *de novo* insertion of a hydrophobic polypeptide stretch into the membrane.

## Topogenic insertion sequences

**Figure 1.**
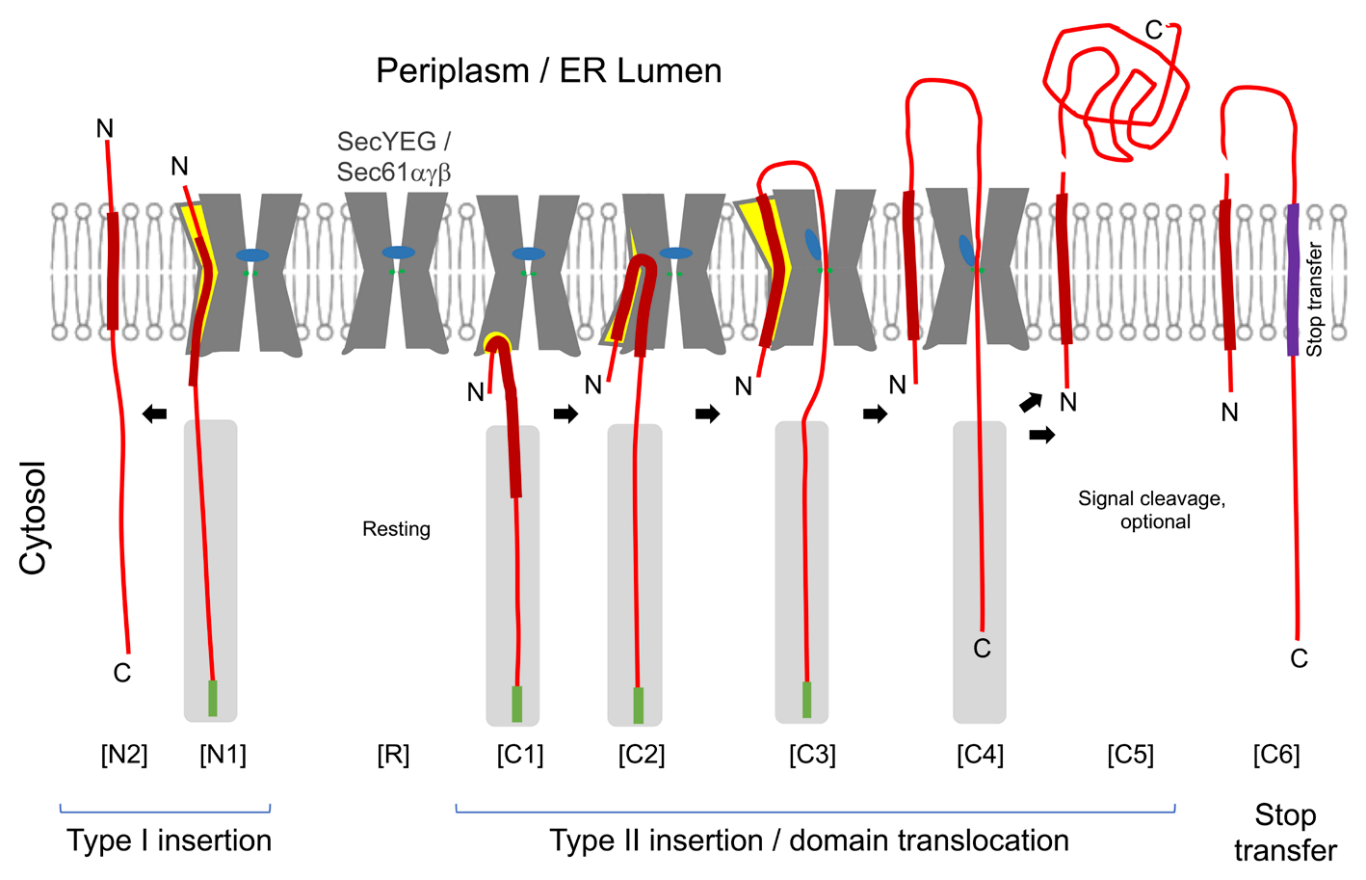
Model diagrams of the Sec-mediated *de novo* insertion of hydrophobic domains. The Sec translocon is schematically depicted to show the polypeptide-conducting channel in the center, the plug helix in blue, and the open lateral gate in yellow. The substrate polypeptide is shown in red, with the thick part representing a hydrophobic segment. R shows the resting (quiescent) state. N1 and N2 show insertion with the C _in_–N _out_ orientation. C1 through C6 shows insertion with the N _in_–C _out_ orientation. Targeting events before insertion could differ depending on the substrates or the organisms. The figure is intended to show the co-translational process with the ribosomal exit tunnel in gray and the tRNA at the growing end of the nascent polypeptide as green rectangles. The timings of plug dislocation and polypeptide enclosure within the channel are shown arbitrarily, as neither has been defined precisely. At least in an early stage of insertion, the plug still occludes the central pore ^5^. Depending on the substrate proteins, the proteolytic cleavage of the signal peptide may or may not take place. Finally, if the second hydrophobic segment (stop-transfer sequence shown in purple) follows, the translocation step halts, and the hydrophobic segment exits the channel laterally to become a type I transmembrane domain, as shown in C6 (the orientation of the stop-transfer segment is type I, but, here, we classify different proteins by the mode of *de novo* insertion of their N-terminal regions). It should be noted that the above diagrams do not take into account the proposal that integration initially proceeds with the N _out_ orientation and reorientation events, later on, accompany the determination of type I versus type II routes ^6^. ER, endoplasmic reticulum.

Topogenic sequences responsible for *de novo* membrane insertion are called signal sequences in the case of secretory proteins and signal-anchor sequences in the case of integral membrane proteins. For this section, readers are advised to refer to Figure 1, which is discussed later in this article in conjunction with the translocon functions. Topogenic sequences invariably contain core sequences enriched in hydrophobic residues, which insert into the membrane to span it with a specific orientation. The C _in_–N _out_ orientation is called type I and N _in_–C _out_ type II (“in” indicates the cytosolic side and "out" the *trans* side of the membrane [see Figure 1]). The orientation is determined by charge characters and length of the flanking hydrophilic regions ^1^.

A canonical signal sequence assumes the type II (N _in_–C _out_) orientation, hence inducing translocation of the mature region that follows it. The signal sequence then receives proteolytic cleavage on the *trans* side by the processing enzyme, signal peptidase, which liberates the mature domain from the membrane. Membrane anchor sequences do not undergo cleavage. Although signal sequences and type II signal-anchor sequences are similar in their membrane topologies, they have different features, such as the length of the hydrophobic core ^7^ and the predicted behaviors in lipidic environments ^8^. Nevertheless, it is useful to assume that these two categories of topogenic sequences share the fundamental principle that underlies their membrane insertion. We point out two observations that support this view. First, leucine-based model sequences can use the common translocon (see below) and function like either a signal sequence or a signal-anchor sequence depending on the length of the leucine stretch and the presence or the absence of the signal peptidase cleavage motif ^7^. Second, in engineered fusion proteins, transmembrane sequences can direct export of the mature domain of alkaline phosphatase ^9^, a bacterial periplasmic protein, even with accompanying cleavage by signal peptidase ^10^.

The C _in_–N _out_ (type I) transmembrane state can be generated by an N-terminal type I signal-anchor sequence as well as by an internal "stop-transfer" sequence that follows a *de novo* insertion signal (see Figure 1). In our view (see below), the stop-transfer sequence does not fall into the category of *de novo* insertion sequence; rather, it depends on the function of the preceding insertion sequence in the upstream region of the polypeptide. Type II *de novo* insertion signals can reside at internal sites such that multiple insertion events in combination with stop-transfer lead to the biogenesis of multi-spanning membrane proteins ^6^. For insertion, the topogenic region of the polypeptide must be targeted to the insertion machinery of the membrane. A typical mechanism is signal recognition particle-mediated co-translational targeting, but we do not discuss targeting mechanisms ^11^ further in this review. Finally, a class of proteins called tail-anchored membrane proteins contains an insertion sequence near the C-terminal end, which must integrate into the membrane with the type II orientation post-translationally ^12^.

## The Sec translocon paradigm must be expanded to account for *de novo* insertion

**Figure 2.**
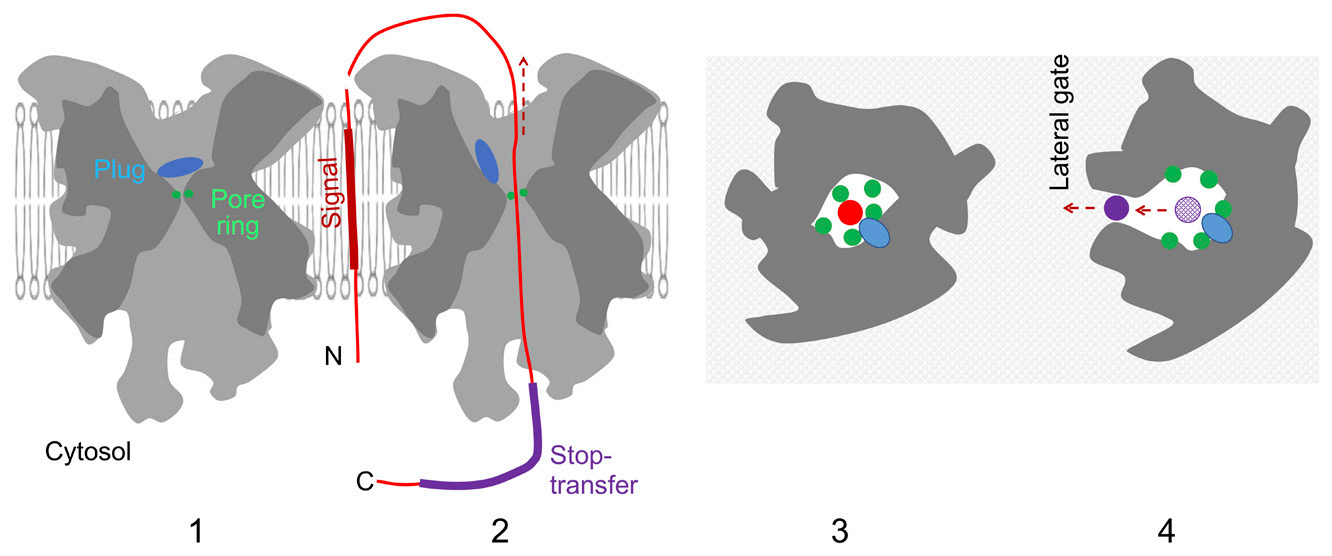
Simplified views of the Sec translocon. Panels 1 and 2 show vertical cutaway images, and panels 3 and 4 show horizontal cutaway images viewed from the trans side. The pore ring, shown in green, is at the constriction of the channel and consists of hydrophobic amino acids that surround the translocating polypeptide (panel 3). The plug, shown in blue, keeps the vertical gate closed in the resting state (panel 1) and is dislocated to open the gate in the working state (panel 2). The translocating polypeptide is shown by the red line (panel 2) or cutaway disc (panel 3). The signal peptide is shown by the thick line (panel 2) in the state already disengaged from the translocon and proteolytically processed. Earlier events of signal peptide insertion into the membrane are shown in Figure 1. The purple line (panel 2) and purple disc (panel 4) show a downstream hydrophobic segment that exits the channel laterally via the open lateral gate (panel 4). This figure was prepared by referring to the structural depiction presented as Figure 1 in Rapoport *et al*. ^19^.

The determination of the Sec translocon structure provided the first look at a polypeptide-conducting channel that allows a substrate polypeptide to traverse the membrane without directly meeting the phospholipid constituent of the membrane ^5, 13– 16^. The conduit is gated by a plug-like helix that seals the channel in the quiescent state ( Figure 2, panel 1), whereas it accommodates a translocating polypeptide in the active, open state with the dislocated plug ( Figure 2, panel 2). The channel is shaped like an hourglass, with a gasket-like constriction at the center, which is formed by hydrophobic sidechains and prevents the leakage of small molecules during ongoing translocation ( Figure 2, panel 3). The translocon is used not only for the complete export of proteins from the cytosol but also for the biogenesis of integral membrane proteins ^13^. For this purpose, the translocon contains the lateral gate, which consists of a few of its transmembrane helices. In a well-known mode of translocon-facilitated integration, called stop-transfer ^6, 17^, a hydrophobic segment of the polypeptide exits the channel laterally to reach the lipid phase of the membrane ( Figure 2, panel 4). This is made possible because the lateral gate of the translocon opens in response to the entry of the hydrophobic segment into the channel, allowing its lateral partition into the lipid phase and acquisition of the C _in_–N _out_ transmembrane configuration ^13, 18^.

This beautiful model of the polypeptide-conducting channel, however, leaves a critical question unanswered: how is the translocating polypeptide accommodated in the channel? *De novo* insertion of an N-terminal polypeptide segment into the membrane will bring the rest of the nascent/newly synthesized polypeptide into the channel interior. *De novo* insertion, which must be induced by the upstream topogenic signal, is a prerequisite for the insertion of a stop-transfer-type hydrophobic segment through the lateral gate into the lipid phase.

In the original publication of the translocon structure, the authors explained that the signal peptide may use a lipid-translocon interface at the lateral gate region to integrate into the membrane ^13^, and recent structural studies support this notion (see below). Probably because the model of the polypeptide-conducting channel is so compelling, however, discussion of membrane integration processes of signal peptides and signal-anchor peptides sometimes starts with an implicit assumption that they reside initially within the polypeptide-conducting channel of the translocon. Recent studies disfavor this assumption (see below). It is of vital importance to elucidate the actual pathways and mechanisms used by Sec translocon for *de novo* membrane insertion of hydrophobic stretches. Before looking into this function of the translocon, we will summarize the importance and the mechanism of actions of the conserved membrane protein biogenesis factors called insertases.

## The YidC insertase provides an intramembrane platform that facilitates membrane insertion of a class of membrane protein

**Figure 3.**
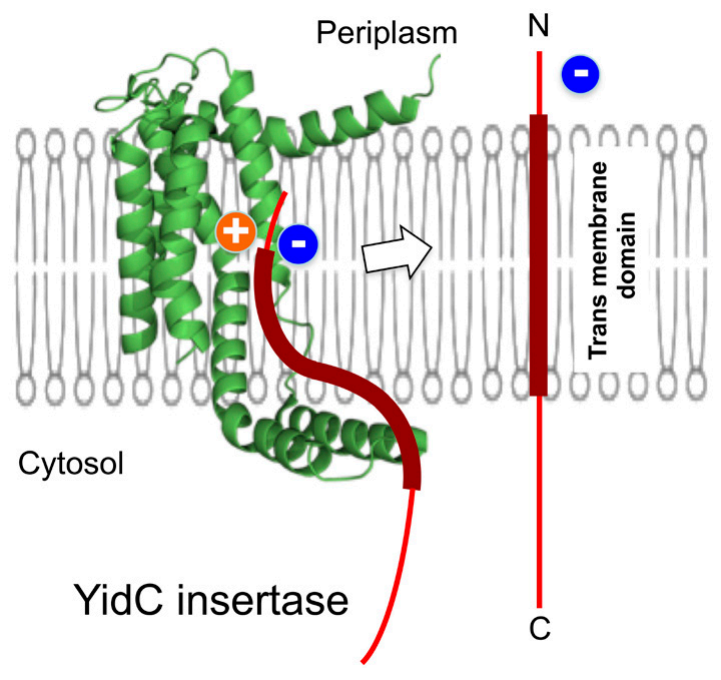
A model diagram of the insertase function. The YidC insertase provides a hydrophilic cavity within the membrane, which a substrate polypeptide uses as the halfway point of insertion ^20^. The figure depicts a possible intermediate state of a class of substrates that have a negatively charged periplasmic tail, which may thereafter be translocated to the periplasmic side, as shown by the arrow, in coordination with the hydrophobic segment partition into the lipid phase.

We point out that the *de novo* integration function of translocon can be viewed as similar in concept to that of “insertases”, which occur in bacteria (the plasma membrane), mitochondria, chloroplasts, and eukaryotes (the ER membrane). Among them, YidC in bacteria is best characterized in its high-resolution structures ^20– 23^ and its insertase function executed independently of the Sec machinery ^24– 26^. YidC facilitates the insertion of a class of simple membrane proteins with one or two transmembrane segment(s) and a small periplasmic (trans-side) domain. Structural, genetic, and biochemical studies suggest that the functional unit of YidC is a monomer ^20, 21, 23, 27, 28^. Its transmembrane helices form an intramembrane cavity that is open toward the cytosol and the membrane interior ( Figure 3); the cavity is embraced by horizontal helices on the cytosolic and the periplasmic sides ^20– 22^. Notably, the cavity is highly hydrophilic and water-accessible, and these features proved to be functionally important ^29, 30^. The presence of an arginine deep in the cavity wall is required for the insertion of a class of substrate proteins with negative charges on the periplasmic tail ( Figure 3). The transient charge attraction is one of the strategies that YidC adopts for the insertase function ^20, 31– 33^, although it is not the exclusive mechanism of insertion ^34^.

In contrast to the translocon channel, which is optimized to accommodate a long and moving hydrophilic polypeptide chain, the insertase does not enclose its substrate ( Figure 3). The current model of the YidC insertase suggests that it provides an intramembrane platform that a substrate polypeptide uses as the halfway point of insertion ^20, 29^. The hydrophilic cavity that is exposed to the lipid phase could cause hydrophobic mismatch problems, which may be alleviated in part by the flexible cytoplasmic loop that covers it ^22^, but could nevertheless disturb the bilayer structure, which might contribute to the insertion mechanism ^30^. The cavity would lower the energy cost of translocation of the short and hydrophilic periplasmic tail by binding to it transiently and circumventing the hydrophobic barrier. This event may (further) disturb the phospholipid organization and lessens the head group barrier against the hydrophobic segment partitioning into the lipidic phase, which will, in turn, drive concomitant translocation of the hydrophilic tail from the halfway point to the periplasmic side ( Figure 3). Circumvention of one barrier or a chain of barriers sequentially could trigger the forward movement of the substrate to the energetically stable, membrane-integrated state. This principle could work for *de novo* insertion of a variety of transmembrane sequences if amphipathic arrangements of the intramembrane platforms are adequately tuned to make them usable as the halfway point. It should be noted that such a platform can be formed dynamically upon access by a substrate protein.

## Insertases as ubiquitous factors in biological kingdoms

Recent studies reveal that eukaryotic cells also possess insertases in the ER membrane. They include Get1/2 and the ER membrane complex 3 (EMC3) subunit of the EMC ^35, 36^. Archaea also have a YidC-like protein ^37^. Eukaryotic Get1/2 mediates the insertion of tail-anchored proteins as an insertase ^12, 38^. The bacterial YidC insertase also facilitates the biogenesis of tail-anchored proteins ^39– 41^. The EMC also functions as an insertase for a subset of tail-anchored membrane proteins ^42^. The EMC is involved in the biogenesis of other types of membrane proteins as well, including multi-spanning membrane proteins with moderately hydrophobic transmembrane regions ^36, 43, 44^. At least in one analyzed case, the EMC acts as an insertase for the insertion of the first transmembrane segment of a multi-spanning membrane protein ^45^.

Both YidC and the EMC have been reported to cooperate with the Sec translocon in assisting the correct biogenesis of membrane proteins that have multiple transmembrane regions. They may contribute to this mode of function by fulfilling chaperone-like roles that facilitate the "folding" of membrane proteins ^36, 43, 46^. However, the division of labor between insertase and Sec in inserting different transmembrane domains could also explain the apparent chaperone function of the EMC/YidC. Specifically, the EMC's ability to facilitate the insertion of the first transmembrane segment of a multi-spanning membrane protein substrate in the correct orientation allows for the correct insertion of the remaining transmembrane regions by the Sec machinery ^45^. The general applicability of such a relay mechanism for insertase–Sec-mediated membrane protein biogenesis remains to be investigated.

## The translocon lateral gate is dynamic in handling hydrophobic topogenic segments of substrates

High-resolution structural information on the Sec translocon in its working state started to accumulate recently through both X-ray crystallography and electron microscopy-based approaches, the latter of which is undergoing remarkable technical progress. Also, efforts are being made to determine structures of translocon that is membrane integrated, rather than after isolation in detergent-solubilized states, by use of lipidic cubic phase crystals ^15^, reconstitution into the nanodisc bilayer ^5, 47, 48^, and cryo-electron tomography of native membranes from cells ^49^. A central point in understanding the roles of the Sec translocon in mediating *de novo* membrane insertion of hydrophobic domains is the roles played by the lateral gate region. A current consensus is that the lateral gate region can accommodate (transiently) the signal sequence or the membrane anchor sequence of either orientation, in its “open” form with a ~22° relative rotation of the N- and C-terminal halves of SecY/Sec61α ^5, 47, 50– 53^, which is also the case for the lateral exit of a stop-transfer-type hydrophobic segment ^54^. The substrate helix enters this region in a manner replacing a "placeholder" helix of the translocon ^51^ and opening the lateral gate toward the lipid phase and also to the trans-side (or the cytosolic side depending on the stages of insertion ^5^) of the membrane ^5, 47, 48, 52, 55– 57^.

While the *de novo* inserting helix should end up in the lipid-embedded state ^58^, the snapshot pictures do not reveal a temporal order of the events in the sequential insertion process. There are crucial questions: when and how is the lateral gate open partially or fully, and when is the plug helix displaced to create a trans-membrane channel? The literature on the timing of lateral gate opening is disparate at a glance, but we can extract one crucial characteristic of the lateral gate: it is mobile and prone to opening to varying extents under different conditions. Structural studies detected a crack on the cytosolic side of the translocon, where some polypeptide moiety from an adjacent molecule resided, possibly as a signal peptide mimic. Substrate polypeptides may use such a crack for the initial recognition and entry step in both the co- and post-translational pathways ^14– 16, 51, 57^ ( Figure 1, C1).

On the other hand, the targeting/driving partners of the translocon, the ribosome or the SecA motor, can induce partial opening of the lateral gate without involving any substrate polypeptide ^16, 59^. Strikingly, cryo-electron tomography studies of the membrane-embedded Sec61–ribosome complex show that the translocon even assumes a fully open conformation in the native membrane without involving any polypeptide substrate ^49^. Also, Sec62/63, the Sec61 partner in the post-translational mode of translocation, causes large lateral gate opening in the absence of substrate ^60, 61^.

A recent study revealed an early intermediate structure of the signal peptide insertion process ^5^, in which nanodisc-integrated SecYEG was complexed with the nascent chain ribosome complex that has a 48-amino-acid-long N-terminal part exposed outside the ribosome. In this structure, the space created by lateral gate helices is open toward the cytosol, while the plug helix still closes the channel. The early intermediate structure differs from the structure determined for the later stage of insertion with the periplasmic-side-open (cytosolic-side-closed) lateral gate accompanied by a ~22° oblique rotation of a set of helices ^51^. Thus, dynamic conformational changes of translocon range from a crack formation on the cytosolic side, through the cytoplasmic opening of the lateral gate, to the full opening of the lateral gate ( Figure 1, C1 to C3). As already mentioned, the dynamic nature of the lateral gate region even allows its full opening before the substrate’s entry ^49, 60, 61^.

## The Sec translocon function to mediate *de novo* membrane insertion can be viewed as insertase-like

With the current knowledge of the insertion process discussed above, it is still unclear how the “passenger” hydrophilic polypeptide following the N-terminal signal is accommodated within the polypeptide-conducting channel ( Figure 1, C4). The plug displacement is a relatively late event, as several structures have been reported in which the plug is not yet displaced in translocon complexes that are already engaged with a substrate or a substrate mimic ^5, 15, 50, 54^. These observations indicate that early events of insertion proceed before the opening of the polypeptide-conducting channel ( Figure 1, N1 and C1–C2). Probably, in *de novo* insertion, substrate polypeptides interact directly with the lateral gate region, which undergoes dynamic remodeling of the constituent transmembrane helices. Although the signal sequence transiently intercalates between some transmembrane helices of the translocon ^51^, its residues also face the lipid phase in the early and later steps of insertion ^5, 47, 48, 52, 55, 56^. We envision that the rearranging lateral gate region provides intramembrane platforms, whose hydropathic properties lower the energy cost of insertion by interacting with the incoming polypeptide. In an extreme case, the strongly hydrophobic substrate might slide along the lipid–translocon interface, as proposed by Cymer *et al.* ^1^, which is supported by thermodynamic considerations ^1^.

The Sec translocon and YidC provide the client polypeptide with thermodynamically similar environments that support membrane protein partition ^62^, suggesting that they share the underlying principles. While the initial crack on the cytosolic side of the translocon forms a hydrophobic patch ^15, 51^, the "primed" lateral gate has a hydrophilic seam ^51^. Upon further opening, the lateral gate cavity will have hydrophobic helices in front ^51^, but it could also have some hydrophilic parts on the wall deep inside ^50^, potentially akin to the YidC cavity. Substrate polypeptides may use the changing hydropathic characters of the lateral gate in their membrane insertion steps. A recent report by He *et al*. ^63^ proposes that two transmembrane segments of Escherichia coli YidC provide a "greasy slide" for a hydrophobic core of a client polypeptide to interact transiently during the insertion process. The insertion of the hydrophobic segment in a loop-like conformation appears to precede the hydrophilic N-tail interaction with the hydrophilic groove of YidC. Thus, an insertase can have multiple intramembrane surfaces of different amphipathic characters, in line with our proposal that the Sec lateral gate could function in similar fashions.

The concept of the polypeptide-conducting channel goes back to the proposal by Blobel and Dobberstein in 1975 ^64^, followed by genetic identification of SecY ^65^ and Sec61 ^66^, biochemical demonstration of their translocase functions ^67– 69^, and, finally, the realization of its structural entity 29 years later by van den Berg *et al*. ^13^. These studies have been influential for our understanding of living cells. We now learn that the translocon must integrate its insertase-like functions and the polypeptide-conduction function to execute the biological task. The thermodynamic principle of membrane insertion suggests the importance of the lipidic constituents of the membrane in *de novo* polypeptide insertion. Indeed, Nishiyama and coworkers have identified a glycolipid molecule, termed MPIase, that is dedicated to protein integration into the membrane in *Escherichia coli* ^70– 72^. The responsible enzyme is conserved in prokaryotic and eukaryotic cells. Now, we are at a stage where studies from various directions merge to enable us to better understand the biogenesis of membrane proteins.
